# Accelerating automatic model finding with layer replications case study of MobileNetV2

**DOI:** 10.1371/journal.pone.0308852

**Published:** 2024-08-22

**Authors:** Kritpawit Soongswang, Chantana Chantrapornchai

**Affiliations:** Department of Computer Engineering, Kasetsart University, Bangkok, Thailand; National Institute of Technology Hamirpur, INDIA

## Abstract

In this paper, we propose a method to reduce the model architecture searching time. We consider MobileNetV2 for 3D face recognition tasks as a case study and introducing the layer replication to enhance accuracy. For a given network, various layers can be replicated, and effective replication can yield better accuracy. Our proposed algorithm identifies the optimal layer replication configuration for the model. We considered two acceleration methods: distributed data-parallel training and concurrent model training. Our experiments demonstrate the effectiveness of the automatic model finding process for layer replication, using both distributed data-parallel and concurrent training under different conditions. The accuracy of our model improved by up to 6% compared to the previous work on 3D MobileNetV2, and by 8% compared to the vanilla MobileNetV2. Training models with distributed data-parallel across four GPUs reduced model training time by up to 75% compared to traditional training on a single GPU. Additionally, the automatic model finding process with concurrent training was 1,932 minutes faster than the distributed training approach in finding an optimal solution.

## 1 Introduction

In traditional deep learning, extensive experiments are necessary to identify proper architectures and hyperparameters. Iterative experiments must be done to explore these choices. For a given architecture, numerous variations are possible, such as adding skipped connections, factoring layers, or increasing the number of layers. Designers must go through these variations to find the best model architecture. It is also time consuming to train each model configuration.

In this paper, we present the acceleration of searching the model architectures. Two approaches are investigated. The acceleration using distributed training and concurrent searching. MobileNetV2 is utilized as a baseline model for the case study with the layer replication with the 3D face recognition task.

In [1, 2], layer replication is one of the approach that can increase the model accuracy without increasing the model parameters. Nevertheless, for a given model, there are a number of possible layers that can be replicated. One may manually try specific layer replications or random layer replications while there is a large search space. To facilitate the experiments, we propose the heuristic for searching the replications. The heuristic can be integrated with either distributed training or concurrent training.

This paper introduces alternative search techniques and utilizes PyTorch’s Distributed Data-Parallel (DDP) module [3] to accelerate the model training process. Batch sizes and learning rates were adjusted properly to optimize the training process. Additionally, we leverage the AsyncIO module [4] to parallelize and distribute the training workload across multiple models concurrently.

Our contribution focuses on the automatic tuning of the MobileNetV2 model architecture to enhance training efficiency. This is achieved through two approaches: 1) distributed training of the model, and 2) concurrent model search under various conditions.

The paper is structured as follows: Section 2 presents necessary related backgrounds. Section 3 presents the methodology and Section 4 presents the experimental results. Section 5 concludes the paper.

## 2 Backgrounds

Due to our focus, this section introduces the background knowledge on convolutional neural networks, 3D face recognition tasks, and distributed training.

### 2.1 Convolutional Neural Networks (CNNs)

Convolutional Neural Networks (CNNs) are a type of deep neural network commonly used for image recognition tasks. They consist of multiple layers, where each layer may or may not be fully connected to the others. Instead of processing the entire image at once, CNNs work on subregions of the input image using mathematical matrices known as convolution filters. These filters are applied to parts of the image to extract features such as lines, curves, patterns, and textures, which are then used in subsequent layers.

In CNNs, the initial layers directly process the basic features of the image, while the later layers aggregate these features into higher-level and abstract representations for classification. This process simulates the human visual system, which focuses on small areas of an image and identifies feature characteristics within those areas.

#### 2.1.1 MobileNetV2

Typically, most deep learning models require lots of parameters; thus, computing and memory capability are necessary to run such a model. In the past, the derived models were deployed on the server-side. With the significant improvement in edge devices, the computing power and camera capabilities have been greatly enhanced. However, deploying machine learning models on these small devices remains challenging due to constraints in processing power and memory capacity. Models must balance high accuracy with efficient resource usage.

**Fig 1:**
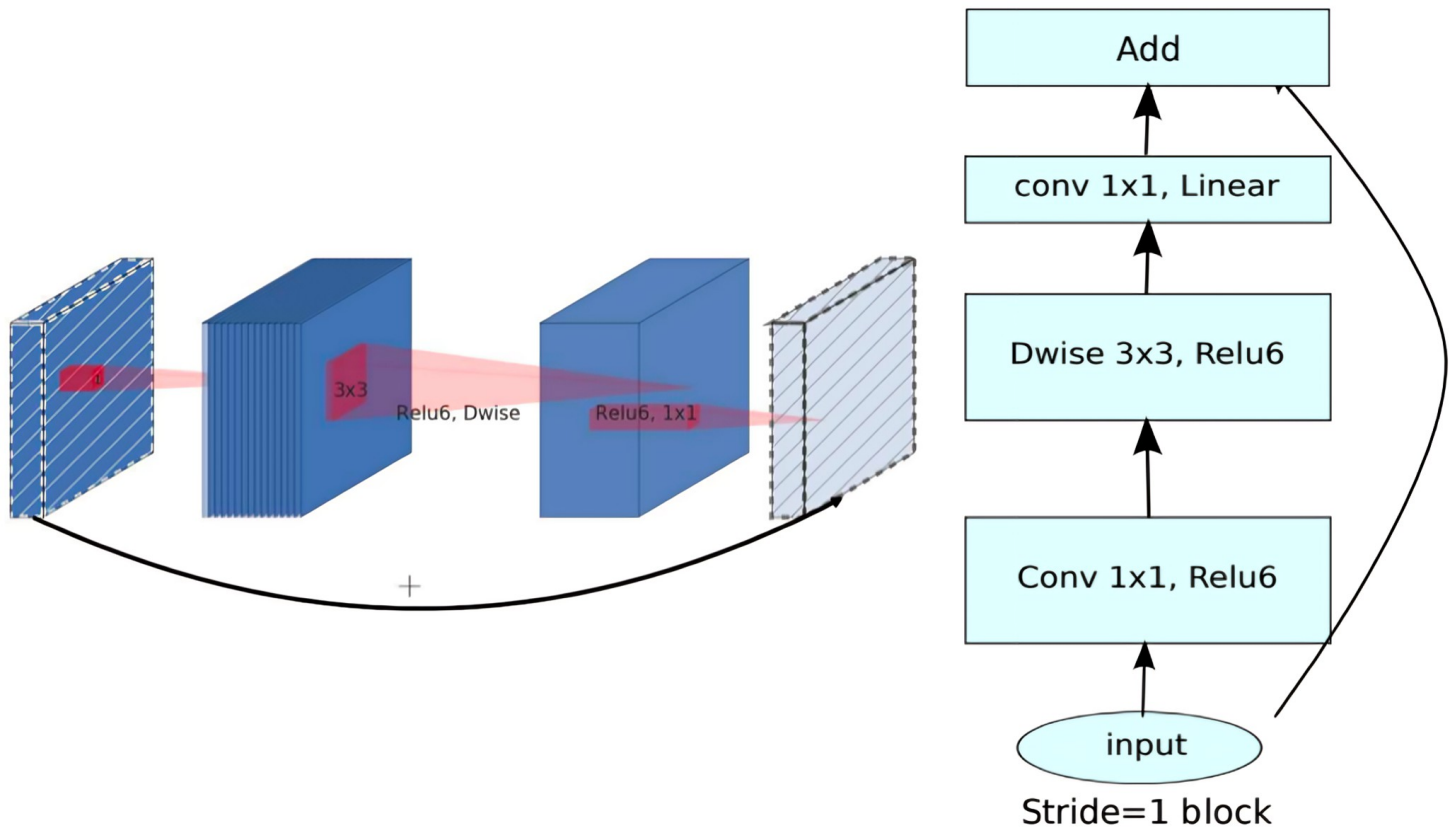
Inverted residual block structure of MobileNetV2.

MobileNetV2 [5] is a type of convolutional neural network (CNN) architecture designed for high efficiency and effectiveness on various mobile devices. Its structure is based on the inverted residual architecture with residual connections between bottleneck layers. Despite its advantages, achieving optimal results for specific tasks often requires network customization. Within this architecture, there are intermediate replication layers used for depth-wise convolution to filter features, resulting in the generation of non-linearity, especially illustrated in Fig 1.

MobileNetV2 consists of a full-scale convolutional layer with 32 filters, followed by 19 remaining bottleneck layers. These layers are designed to separate and transform various feature characteristics, enabling the network to learn complex patterns in input data and make predictions based on these patterns. MobileNetV2 is widely adopted in mobile applications due to its performance across various tasks, as well as the compact model size and low computational requirements.

#### 2.1.2 Position Map Regression Network (PRNET)

**Fig 2:**
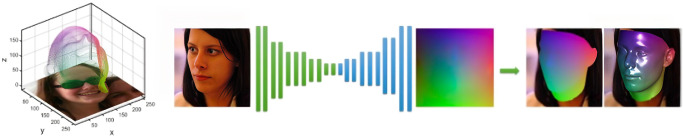
PRNET [6].

Position Map Regression Network (PRNET) [6] is a lightweight Convolutional Neural Network (CNN) with the capability to generate 3D facial structures from 2D images. This is achieved by utilizing a 2D representation called the UV position map, which records the complete 3D facial shape on the UV position map. The network is trained to regress the UV position map, which contains full 3D information and skin tightness adjustments, to produce the desired output, as shown in Fig 2.

PRNET performs effectively under various conditions, including changes in facial pose, lighting, and occlusion. Furthermore, it excels in real-world scenarios and operates faster compared to alternative methods.

#### 2.1.3 Multi-Task Cascaded Convolutional Neural Network (MTCNN)

Multi-task cascaded Convolutional Neural Network (MTCNN) is an algorithm for detecting and positioning faces in images [7]. MTCNN has extensive applications in various tasks, including face detection, identifying key facial landmarks, and face alignments. It is structured as a cascaded sequence of three interconnected convolutional neural networks to detect and align faces in images.

The first CNN generates multiple bounding boxes, the second fine-tunes the bounding boxes and estimates the positions of key facial landmarks, and the third further refines the positions of these landmarks and aligns the face within the image.

### 2.2 Distributed training

Distributed training is a technique used for training large and complex neural networks by distributing the computation of large datasets across multiple computational units or GPUs to accelerate the training process. Distributed training can be primarily categorized into two main types: data parallelism and model parallelism.

#### 2.2.1 Data parallelism

Training with data parallelism is a common type of distributed training. This process typically starts by creating processes equal to the number of available GPUs on the server or distributed across machines in a network. The model is then replicated to prepare for training within each process. Afterward, the entire dataset is divided and distributed for training the model in each GPU.

**Fig 3:**
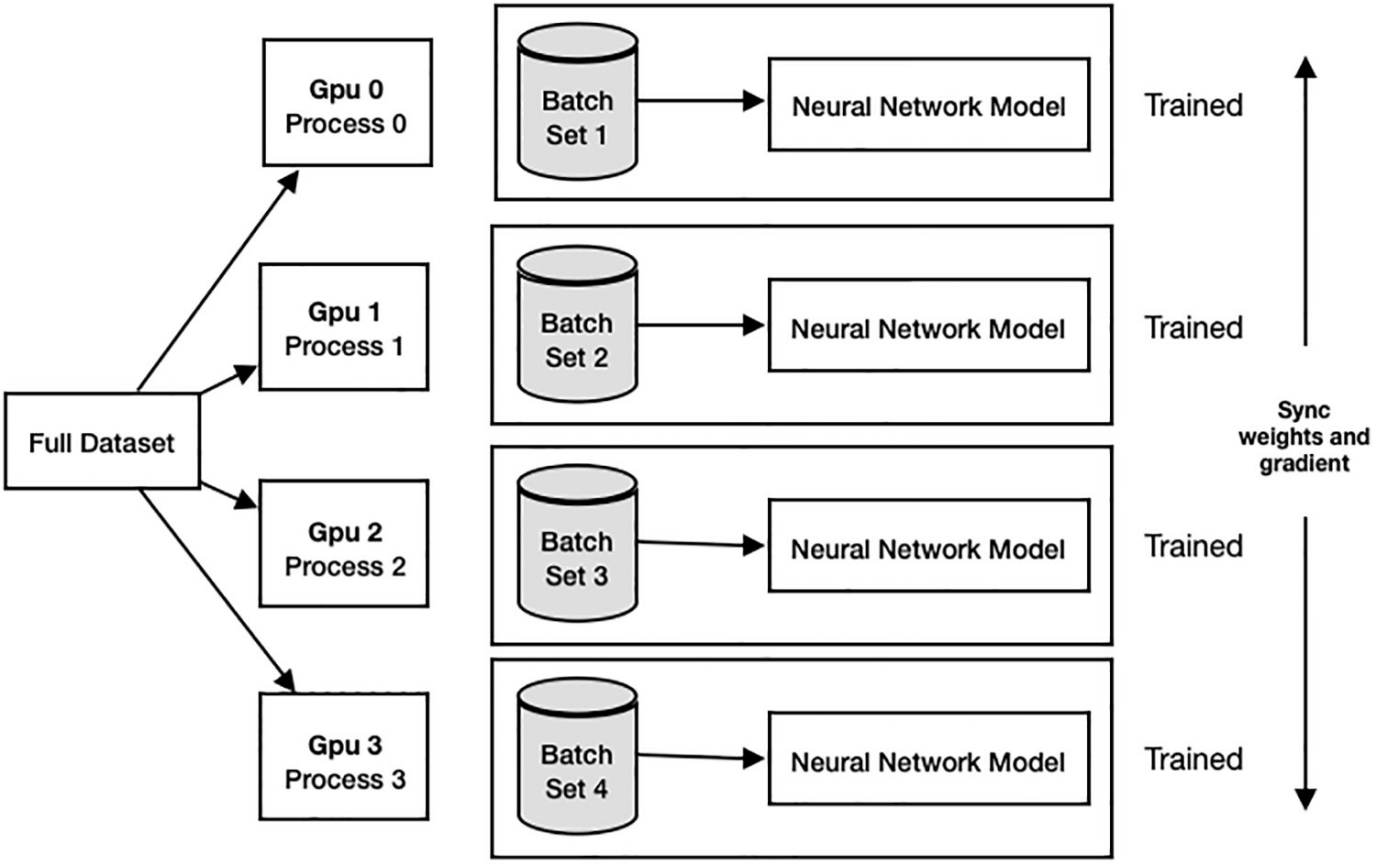
Data parallelism.

When training begins, each process independently learns from its assigned data. When completing the training iteration, the processes gather the gradients from each instance and update the model parameters for the use in subsequent rounds of training. Each process is responsible for a separate, non-overlapping set of GPU resources. (as shown in Fig 3).

#### 2.2.2 Model parallelism

**Fig 4:**
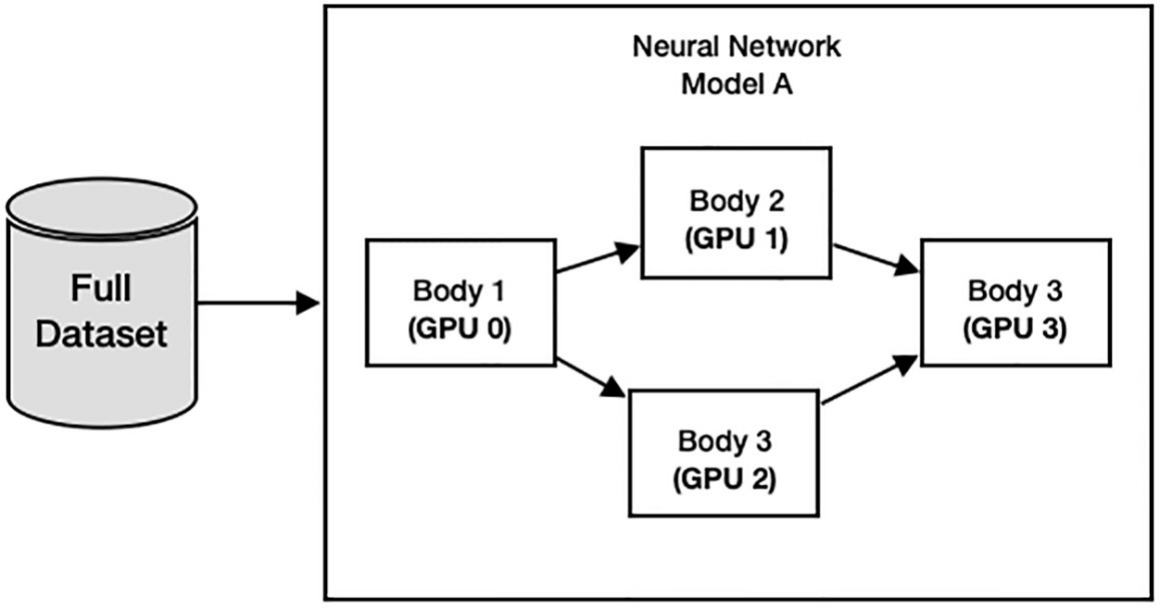
Model parallelism.

Model parallelism in distributed training focuses on allocating portions of the model to each GPU to efficiently enable large models to work with limited resources. This allows the large model to work efficiently on constrained resources in Fig 4.

Currently, there are various frameworks available that support distributed deep learning. Common ones include TensorFlow [8] and PyTorch [9]. Research has shown that distributed deep learning using PyTorch can significantly improve performance [3, 10].

### 2.3 Related work

This subsection presents previous researches which are related to our work: Deep learning architecture search, 3D face recognition tasks, and distributed training.

#### 2.3.1 Deep learning architecture search

It is well-known that finding the suitable architecture for deep learning tasks required lots of experiments for finding a suitable architecture and hyper-parameter tuning. Different tasks need different network architectures and different parameters. Neural Architecture Search (NAS) becomes an emerging research to resolve such obstacles. Several types of NAS were proposed based on one-shot, zero-shot, a few-shot learning etc. [11] Some combined zero-shot and one-shot approaches and utilized weight-sharing [12]. These works attempted to reduce search space and utilized existing information to improve the training iteration time.

In [13], comprehensive insights of various search strategies and performance estimation methods showed the potential of NAS in optimizing neural network architectures. This is supported by the work of Liu et al. [14], who introduced Differentiable Architecture Search (DARTS) as a method for efficiently finding optimal CNN architectures through continuous relaxation of the architecture representation.

Note that these works focus on the search to find the general architecture by exploring combination each type of convolution layer types and hyperparameters while our works demonstrate the exploration of specific type of layer operations. Particularly, we consider the operations of layer replications and the heuristic to search the proper replications to improve the overall accuracy.

#### 2.3.2 3D face recognition tasks

In 3D face recognition tasks, the face landmarks’ depth were considered to improve the accuracy of prediction. For such a task, additional depth map can be considered as another input to the network.

Donghyun et.al. introduced a method for Deep 3D face recognition using deep learning models and 3D data augmentation techniques [15]. The model employed a VGG-style neural network to extract feature maps from 3D facial landmarks. The data augmentation techniques encompassed facial pose variations, random patch sampling, and emotion expression synthesis. Changes in facial pose were applied to address the challenge of non-optimal convergence in rigid ICP registration, requiring the creation of transformation matrices for 3D facial landmarks. Data augmentation through random patch sampling involved generating 18x18 patches on the 2D depth map to prevent overfitting in specific facial regions. Furthermore, additional data was augmented through emotion expression synthesis.

Ying Cai et.al. [16] presented a deep learning approach for 3D face recognition. Their methodology consisted of three primary steps: 1) Utilizing a fast pre-processing method before 3D scanning. 2) Applying Principal Component Analysis (PCA) for rapid and accurate adjustment of the nose tip to the surface points in the 3D space. 3) Projecting 3D scanned images onto 2D images at a specific distance and normalizing them using just three facial landmarks. This enhancement improved the usability of 3D face recognition in the realm of augmented reality. Additionally, the researchers employed various data augmentation techniques, including 3D rotation, cropping, zooming, and increasing detail to enhance the efficiency of their deep learning model. The model was constructed using different deep neural network architectures, and they asserted that their model achieved high accuracy.

Recently, Romphet et al. [1] proposed a method for 3D face recognition by modifying the model’s input layer to receive 3D images along with RGB images on various state-of-the-art models. They also introduced an automatic model modification method with layer replication for MobileNetV2 to enhance its performance. The model was evaluated using a dataset generated by Generative Adversarial Networks (GANs).

#### 2.3.3 Distributed training

For large training data, each epoch can take longer time for al GPU with small memory since the data need to split into several small batches. With multiple GPUs and data parallelism, the large batch size can speed up the training iterations.

Li et al. [3] provided an in-depth analysis of PyTorch’s Distributed Data Parallel (DDP) module, demonstrating its effectiveness in parallelizing CNN training across multiple GPUs. They highlighted key techniques such as gradient bucketing, overlapping communication with computation, and skipping synchronization to enhance performance and scalability. Goyal et al. [10] discussed strategies for large-scale distributed training, emphasizing the importance of optimizing communication and computation overlap.

Korn et al. [2] enhanced the work in [1] where they focused on model input layers and automatic model modification with layer replication. The authors proposed an automatic model modification with layer splitting to increase the model’s performance. The distributed data parallel techniques were utilized to decrease the training time.

In this paper, the algorithms proposed by Romphet et al. [1] and Korn et al. [2] were considered. Additionally, we revised, illustrated, and elaborated on the methods outlined in these works to enhance clarity. Furthermore, we introduced a novel alternative: automatic model finding during concurrent training to improve the performance of the automatic model finding method when compared to the distributed training approach.

## 3 Methodology

This section provides details about the equipment used in the experiments, the datasets and data preparation, the types of models used in the experiments, and the experimental procedures.

### 3.1 Hardware and device

The experiments were conducted on servers located at Kasetsart University, Bangkhen, Thailand. Two servers were utilized, each equipped with 256 GB of RAM, Intel(R) Xeon(R) Gold central processing units (CPUs) running at 2.10 GHz, and NVIDIA Tesla V100-SXM2 32GB graphics processing units (GPUs). The first one was configured with 4 GPUs, while the second server had 2 GPUs.

### 3.2 Data gathering

The facial dataset used in this experiment was obtained from an international competition held in 2019 by the Bench Council International Artificial Intelligence System Challenges. The dataset consists of a total of 403,067 facial images from 1,208 individuals [17].

### 3.3 Data preparation and data preprocessing

**Fig 5:**
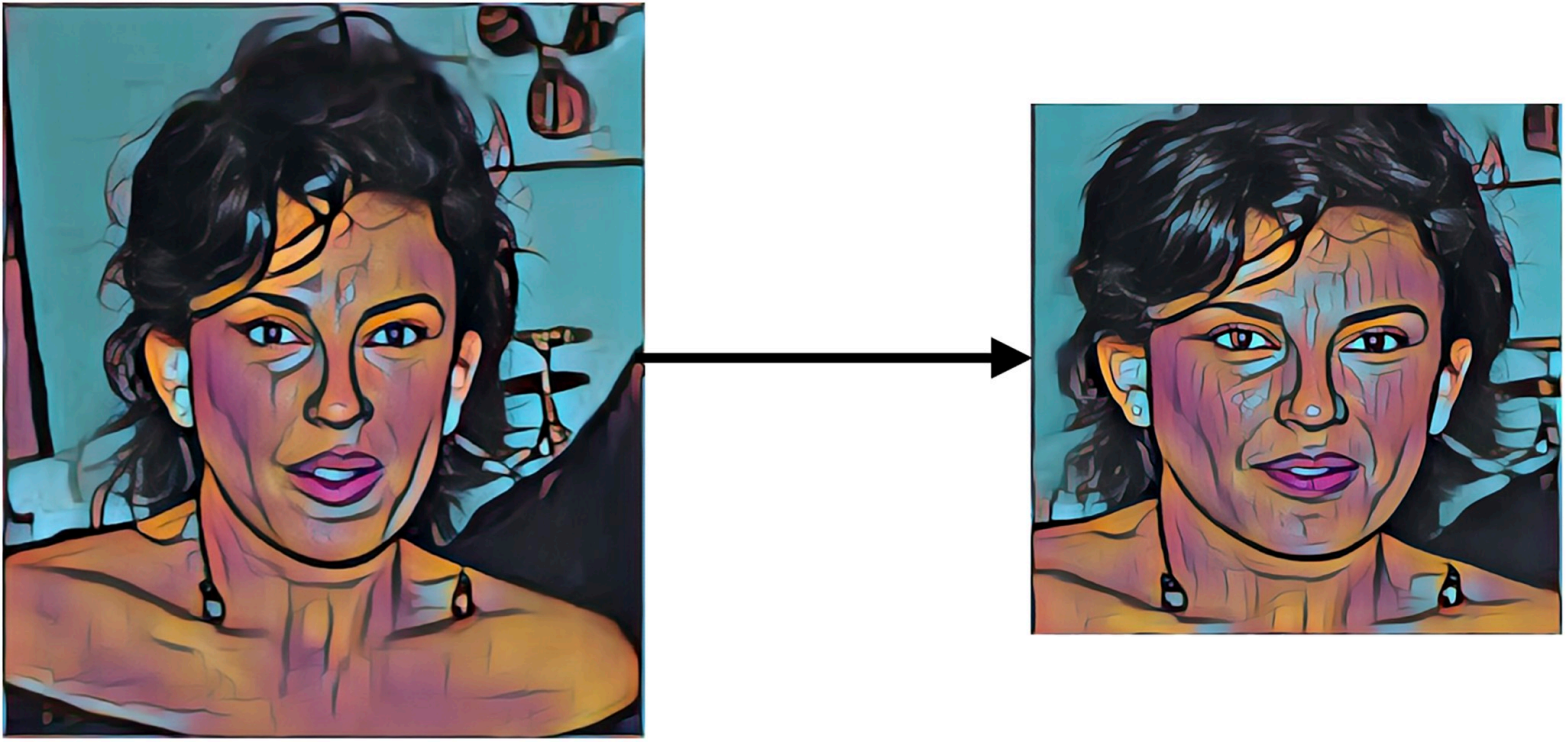
Cropping and aligning image.

To prepare the images for experimentation, the initial step involves detecting and aligning facial components on the images in a consistent manner. This process is accomplished using MTCNN. Once the bounding boxes of facial positions are identified, the images are resized to 224x224 pixels (as shown in Fig 5).

**Fig 6:**
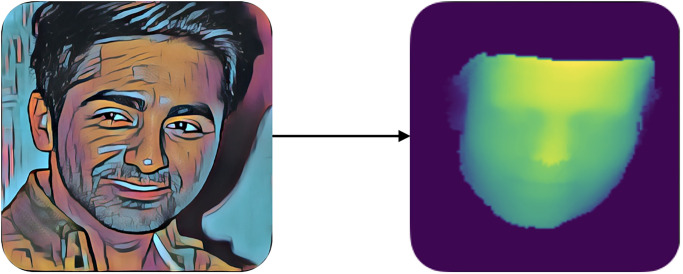
Generating depth image from RGB image.

After that, we created the 3D facial images (depth images) from the prepared 2D facial images. This is achieved using PRNET, as shown in Fig 6.

**Fig 7:**
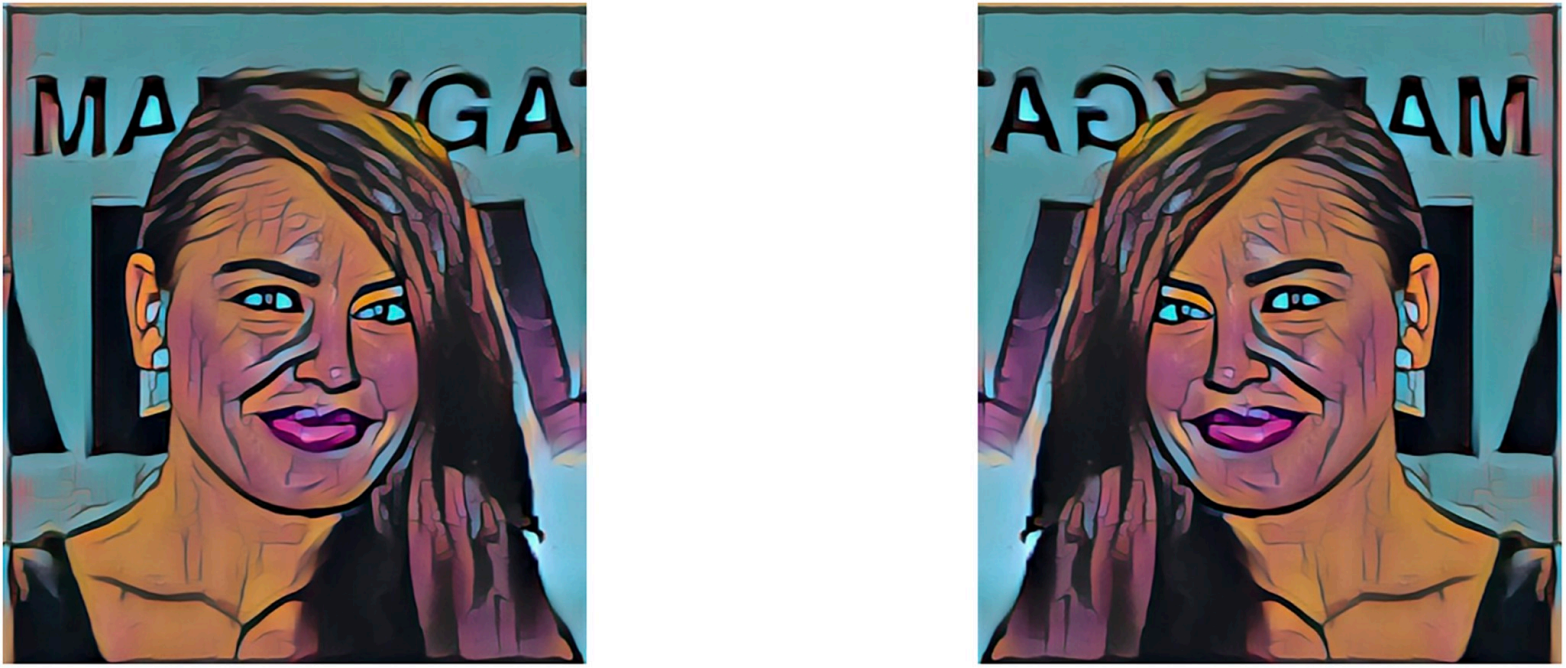
Example of horizontal flipping.

Before inputing the images into the model, we employ a technique, random horizontal flipping, as shown in Fig 7. This is done to increase the diversity of the data and enhance the model’s performance. By learning from images with objects or planes in different orientations, the model gains the ability to recognize objects or planes with greater variations in their arrangements.

### 3.4 Model overview

To investigate the different ways of using depth to enhance the accuracy, we studied three different models, each with distinct input layers, namely, RGB, RGBD, and RGB+D. All three models share a common foundation based on MobileNetV2 architecture.

#### 3.4.1 RGB input layer

Using RGB as the input data is a common approach for image data, as typical images have three color channels (Red, Green, and Blue). Therefore, the convolutional neural network (CNN) layers can accommodate the three-channel data. This model is considered as a baseline in the experiments.

#### 3.4.2 RGBD input layer

**Fig 8:**
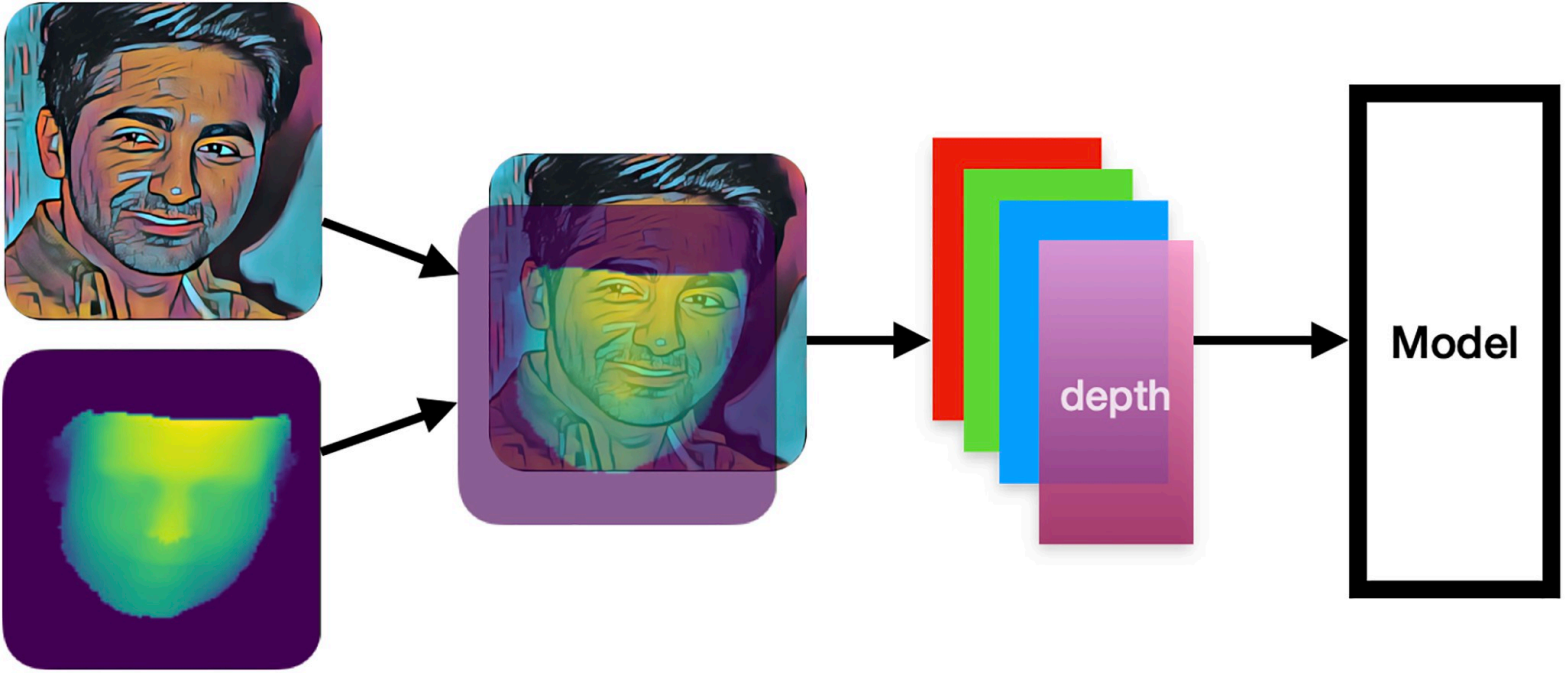
RGBD input layer.

When using RGBD data as input, the RGB image with three data channels is combined with the depth image with one data channel. This results in the input data with a total of four channels for the model (as shown in Fig 8). The number of channels becomes 4.

#### 3.4.3 RGB+D input layer

**Fig 9:**
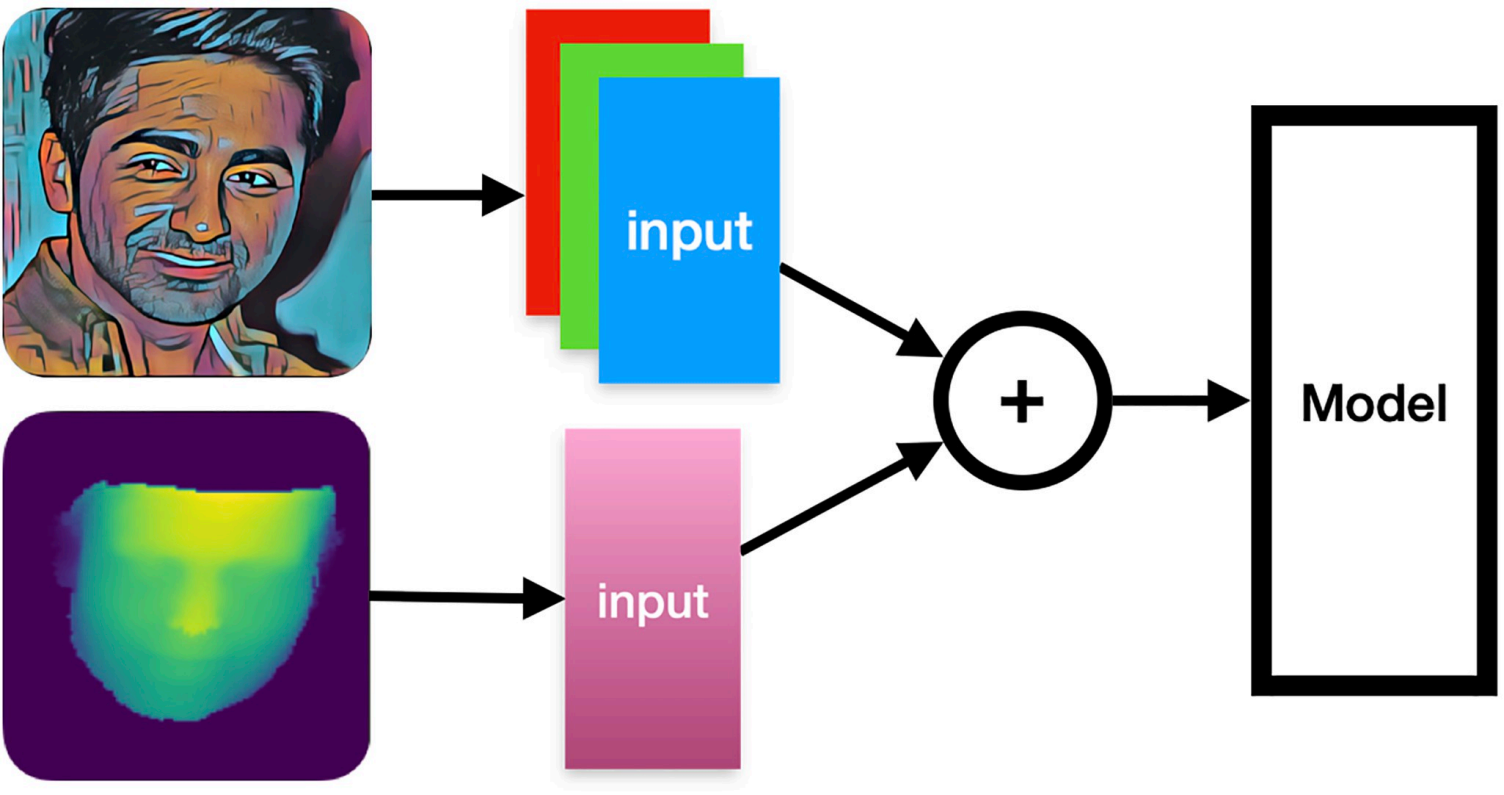
RGB+D input layer.

The RGB+D input data is processed by two distinct data channel handling layers, which are explicitly split. It consists of two layers: the RGB data channel handling layer and the depth data handling layer. The data outputs from both of these layers are summed, and the results are fed into the model, as shown in Fig 9.

### 3.5 Experiment configuration

Since we also investigate the effect of distributed training, the model training was conducted in two configurations: the traditional single-CPU/GPU model training and distributed data training across four GPUs.

For the traditional model training, a batch size of 16 and a learning rate of 0.001 were used, and the training was set for a total of 50 epochs. EarlyStopping was employed during model training when performing an automated search for the best model, reducing training time. PyTorch library was used, which includes the DistributedDataParallel (DDP) module to facilitate distributed data training.

When distributed data training was utilized to accelerate the training process and reduce the overall training time, we adjusted hyperparameters, such as batch size and learning rate, significantly influenced the training efficiency, speed, and accuracy of the model. Too large batch size can speedup the training while degrading the accuracy [18]. Proper batch size should be investigated to achieve the best accuracy. We increased the batch size to 128 and used the learning rate of 0.1, resulting in a substantial speed-up in model training while maintaining effectiveness.

### 3.6 Automatic model finding with layer replication

This section explains the principles used for automatic model discovery, which include layer replication, weight transferring, and two methods for automatic model discovery: automatic model finding on distributed training and automatic model finding on concurrent training of multiple conditions. Both of these methods use early stopping to reduce training time and utilize RGB+D as the input data.

#### 3.6.1 Layer replication

**Fig 10:**
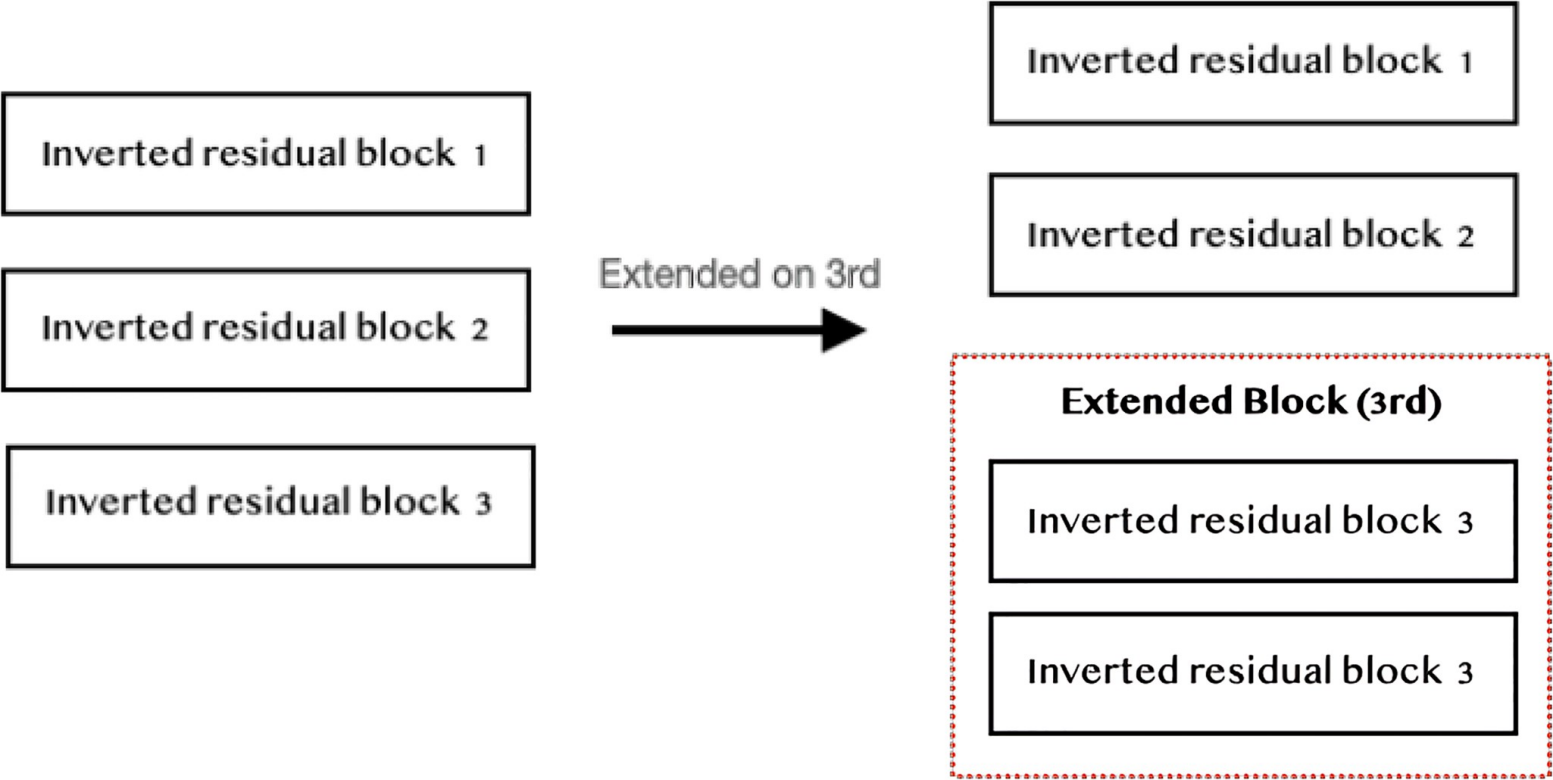
Example of extending the block (1).

MobileNetV2 model consists of a series of Inverted Residual Blocks arranged in a sequence. Some of these blocks have the same input and output data sizes, allowing us to expand the model without any issues by adding additional blocks after the existing ones (as shown in Fig 10). This increases the depth of the model and enables it to capture more complex patterns in images, which can increase the model accuracy without increment of parameters. While training, the small accuracy improvement may not be worth if the training time is much longer. However, in some of the cases, we may be interested in the best model since we will take the model for deployment. Or if the training resource is limited, we can early stop the training and proceed with other replication configuration.

**Fig 11:**
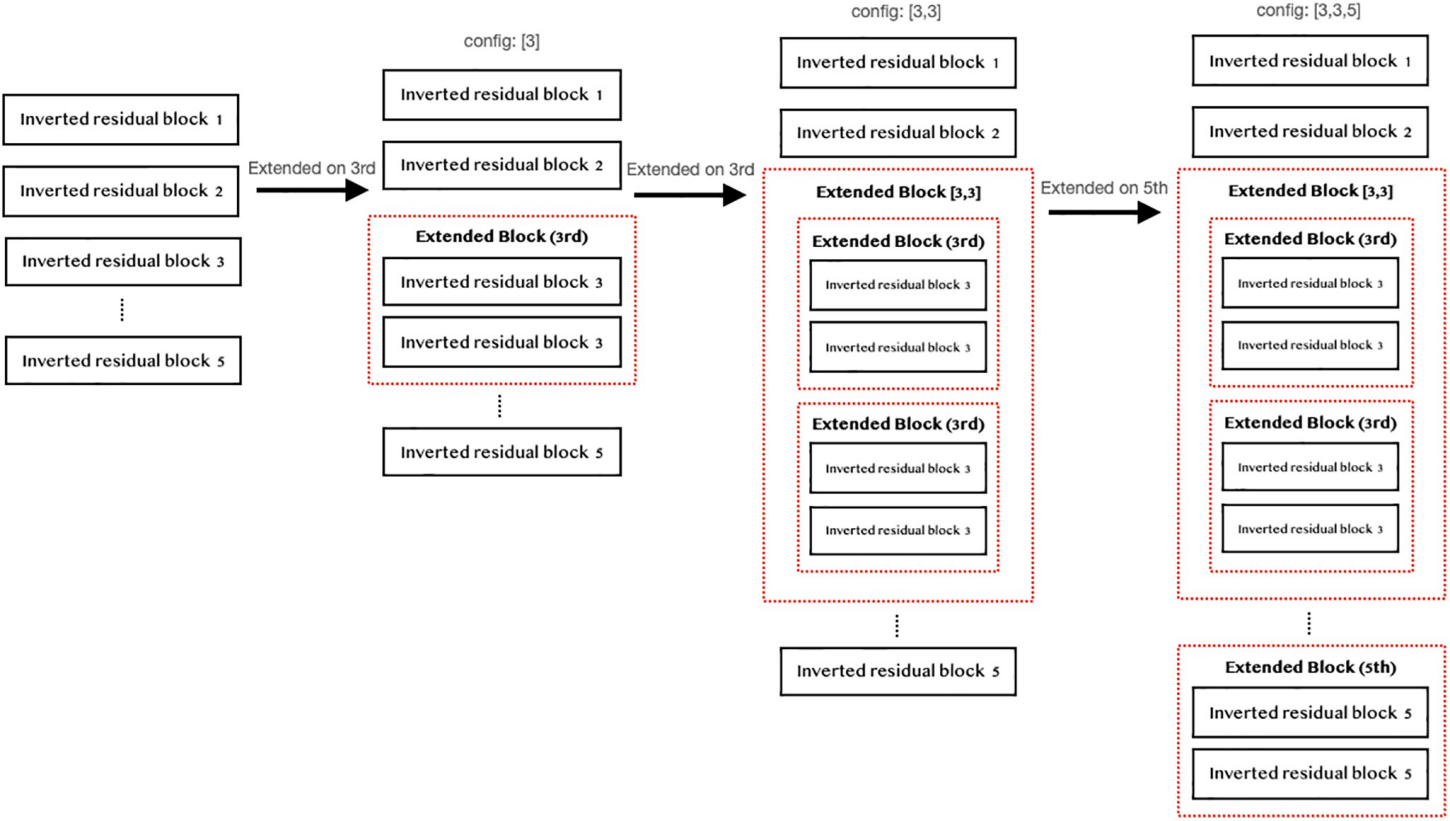
Example of extending the block (2).

For this paper, specific block positions are used, namely the positions [3^rd^, 5^th^, 8^th^, 13^th^, 15^th^]. By expanding the layers, we can set the replication positions at multiple locations. For example, setting the layer replication as [3, 3, 5] means 1) expanding layer 3 in the model, 2) expanding layer 3 in the model again, and 3) expanding layer 5 in the model (as shown in Fig 11).

#### 3.6.2 Weight transferring

**Fig 12:**
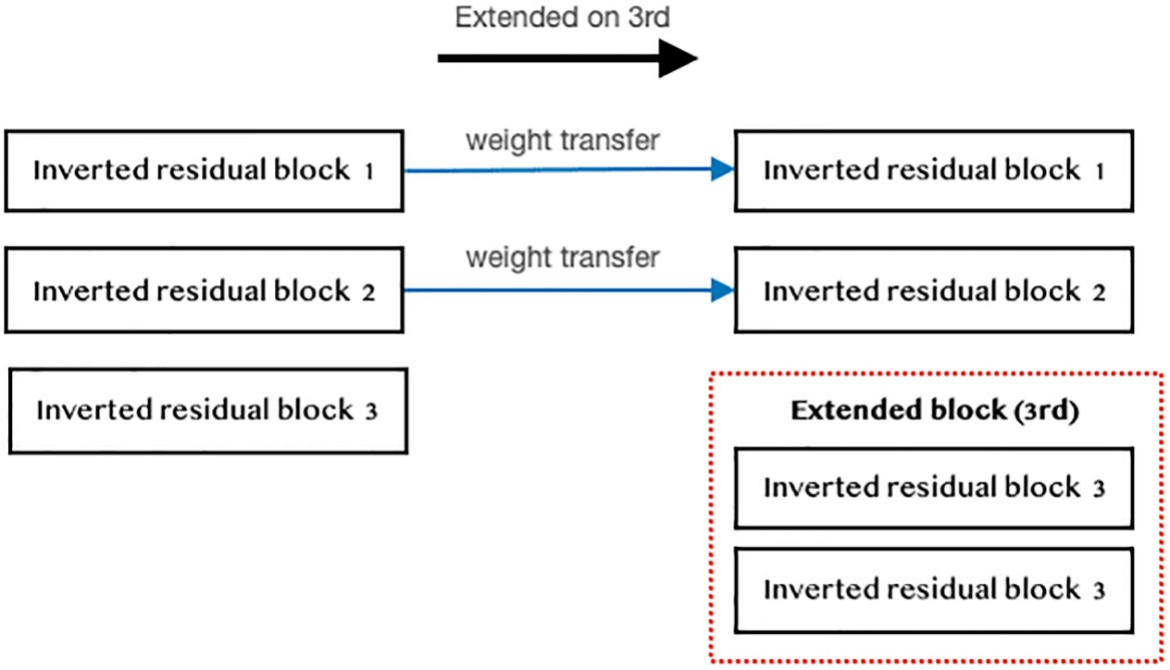
Example of weight transferring.

During the weight-transferring process, we transfer the model weights from the model that hasn’t undergone layer replication to the model whose layer has been expanded. Every block in the model that has been expanded will obtain the weights, except for the newly expanded blocks (as shown in Fig 12). Weight transferring is employed when working with both a pre-trained model and a model with newly expanded layers. It allows the new model to inherit weights from the pre-trained model, except for the newly expanded blocks that get randomly initialized. This enables the new model to learn more efficiently from the pre-trained model and saves time during model training.

#### 3.6.3 Automatic model finding on distributed training

This section explained the process of automatic model search during distributed training. In our experiment with MobileNetV2 model, there are a total of 5 positions where Invert Residual Blocks can be expanded: [3^rd^, 5^th^, 8^th^, 13^th^, 15^th^]. The automatic search process starts with model creation and layer replication, beginning with block 3. The model is then trained with the expanded layers in block 3. After training, the accuracy of the model with expanded block 3 is recorded. The process is repeated in the sequential order from block 5^*th*^ to block 15^*th*^.

Once the models with all possible layer replications have been trained, the one with the highest accuracy is selected for weight transfer. The configurations of the selected models are stored, including the positions of the most accurate layer replications. These configurations serve as the base configuration for the next search iteration.

For example, in the first iteration, it is found that the most accurate layer replication position is the config = [5]. In the next round, the process starts by creating a model with layer replication at block position 3 following the base configuration. The expanded layer configuration becomes [5, 3], and the model is trained. This process continues, incrementing the layer replication positions from [5, 5] to [5, 15].

**Fig 13:**
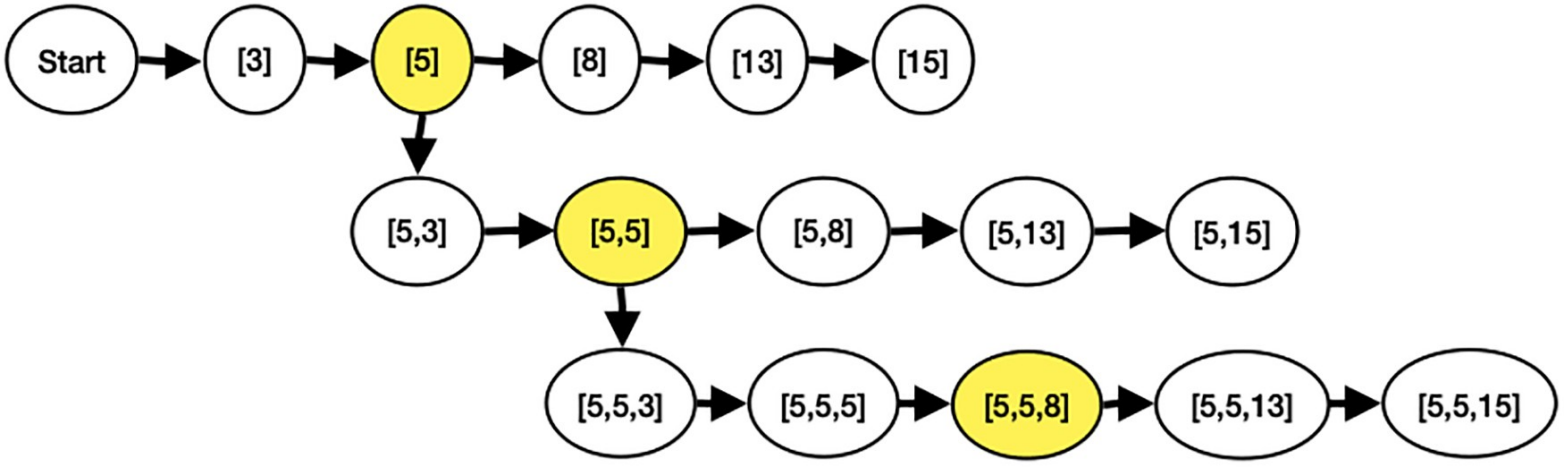
Example of the workflow of automatic model finding on distributed training.

**Fig 14:**
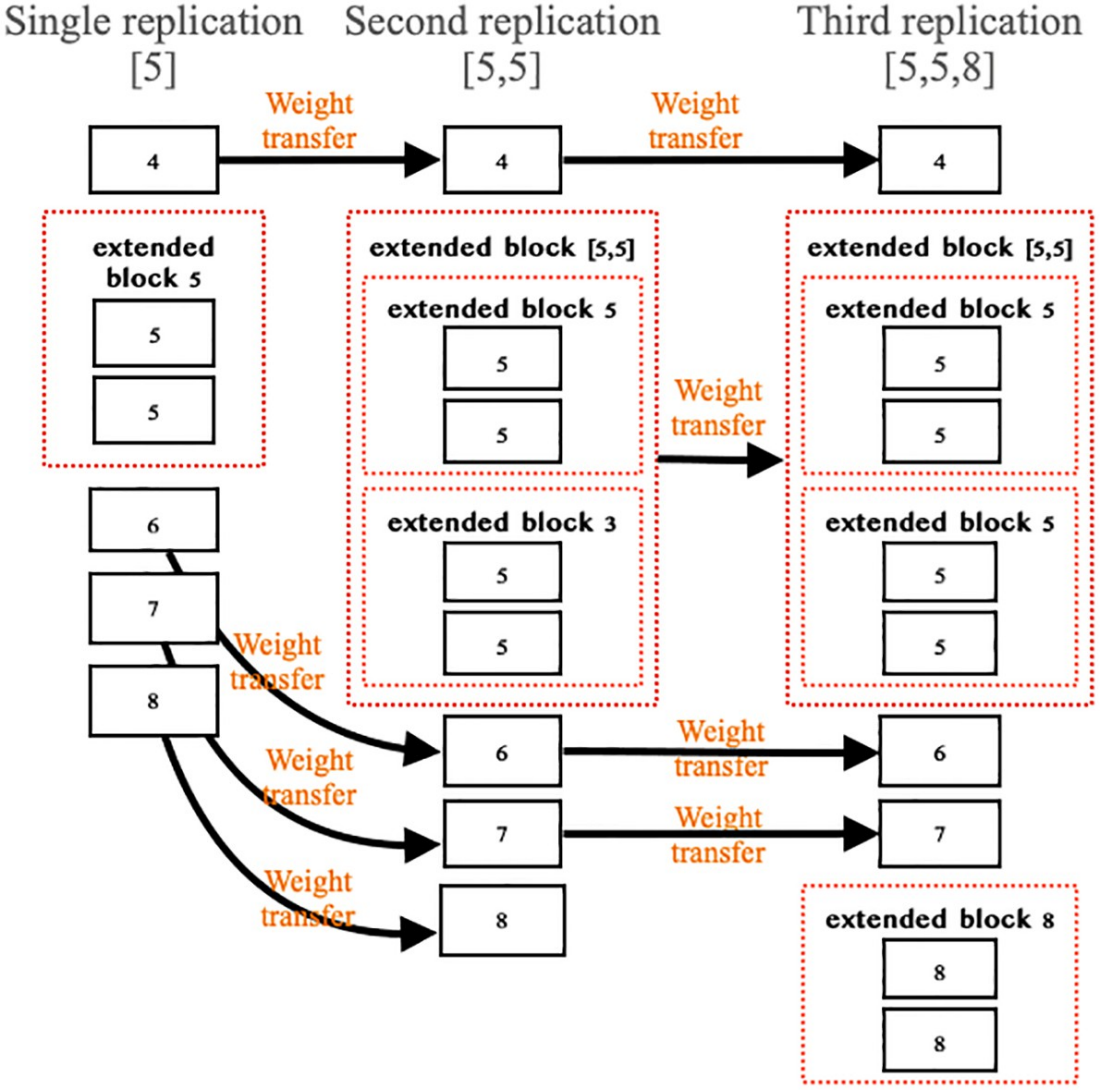
Example of automatic model finding on distributed training.

After training all possible models, the automatic search compares the accuracy difference between the best model of the current round and the previous round. If the difference is greater than the stopping threshold, the next search round begins. This iterative process continues until the accuracy difference between rounds is less than the stopping threshold, as shown in Figs 13 and 14.

The algorithm for automatic model search on distributed training can be summarized in Algorithm 1.

#### 3.6.4 Automatic model finding on concurrent training

The process of conducting an automatic model search on concurrent training across different conditions follows a similar strategy to the distributed training automatic model search with MobileNetV2 model blocks extendable across five positions. The method involves adjusting a single model within the original or single-GPU training setup, executed on two or more machines, each of which has multiple GPUs. In our case, we used two machines equipped with 4 GPUs and with 2 GPUs respectively.

Each extended model at the 3^rd^, 5^th^, 8^th^, and 13^rd^ layer replication positions are trained on the server with 4 GPUs, with each GPU responsible for training each model in sequence. The model expanded at the 15th layer position is trained on the server with 2 GPUs, specifically on GPU:0. Once every model with distinct layer replication positions completes training, the automatic search selects the model with the highest accuracy at each position to serve as the base configuration for layer replication settings and weight transfer in the subsequent search iteration.

**Fig 15:**
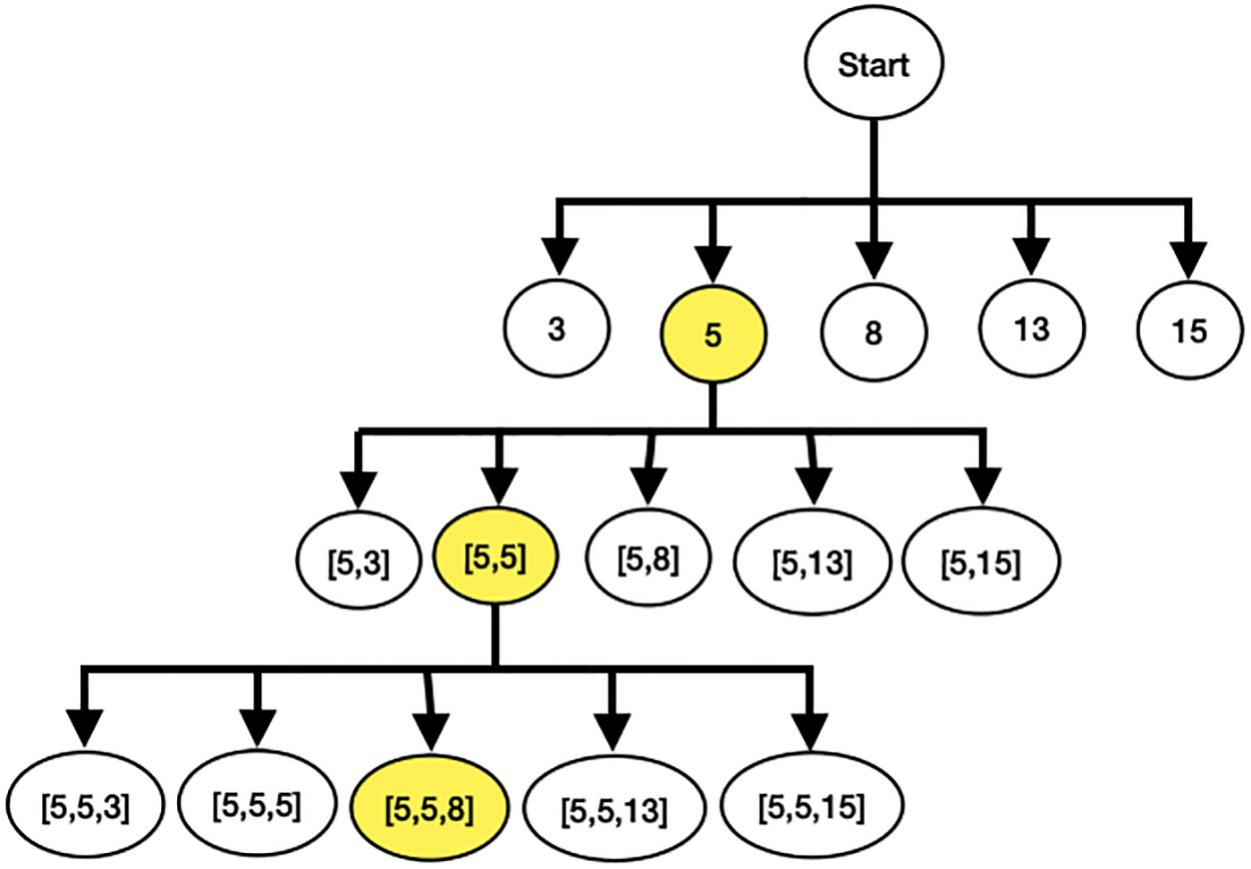
Example of the workflow of the automatic model finding on concurrent training.

If the accuracy difference between the best-performing model in the current round and the previous round exceeds the stopping threshold, a new search round begins until the accuracy difference falls below the stopping threshold (as depicted in Fig 15). Leveraging Python’s Asyncio library enables the concurrent training of the various model configurations.

**Algorithm 1**: Automatic Model Finding On Distributed Training

**Data**: *allow*_*replicate*_*layers*: list, *best*_*replicated*_*config*: list, *best*_*config*_*in*_*round*: list, *best*_*model*_*path*: string,

**while** |*max*_*acc*−*max*_*acc*_*in*_*rep*_*round*| > *threshold* **do**

 **if** *max*_*acc* < *max*_*acc*_*in*_*rep*_*round* **then**

  *max*_*acc* = *max*_*acc*_*in*_*rep*_*round*;

 **end**

 *layer*_*config* = *best*_*replicated*_*config*.copy();

 **for** *i* ***in*** *allow*_*replicate*_*layers* **do**

  *config* = *layer*_*config* + [*i*];

  *acc*, *path* = *trainmodelDDP*(*config*, *best*_*model*_*path*);

  **if** *acc* > *max*_*acc*_*in*_*rep*_*round* **then**

   *max*_*acc*_*in*_*rep*_*round* = *acc*;

   *best*_*model*_*path* = *path*;

   *best*_*replicated*_*config* = *config*;

  **end**

 **end**

**end**

**Function** *trainmodelDDP*(*replicated*_*layers*, *path*):

 *model* = *createmodel*(*replicated*_*layers*);

 **if** *path* **then**

  *previous*_*model* = *load*_*model*(*path*);

  *model* = *weight*_*transfer*(*previous*_*model*, *model*);

 **end**

 train *model* (Distributed Data parallel across 4 GPUs), return (*accuracy*, *path*);

The algorithm for automatic model search on concurrent training can be summarized in Algorithm 2. The control variables used in both algorithms are summarized as follows:

**Algorithm 2**: Automatic Model Finding On Concurrent Training

**Data**: *allow*_*replicate*_*layers*: list, *best*_*replicated*_*config*: list, *best*_*config*_*in*_*round*: list, *best*_*model*_*path*: string,

**while** |*max*_*acc* − *max*_*acc*_*in*_*rep*_*round*| > *threshold* **do**

 **if** *max*_*acc* < *max*_*acc*_*in*_*rep*_*round* **then**

  *max*_*acc* = *max*_*acc*_*in*_*rep*_*round*;

 **end**

 *layer*_*config* = *best*_*replicated*_*config*.copy();

 *config*_*gpu*0, *config*_*gpu*1 = *layer*_*config* + [3], *layer*_*config* + [5];

 *config*_*gpu*2, *config*_*gpu*3 = *layer*_*config* + [8], *layer*_*config* + [13];

 *config*_*gpu*0_*ssh* = *layer*_*config* + [15];

 *command*_*run* = [*trainModel*(*config*_*gpu*0, 0), …, *trainModel*(*config*_*gpu*0_*ssh*, 0)];

 *result* = *Asyncio*.*run*(*command*_*run*);

 *max*_*acc*_*in*_*rep*_*round*, *best*_*model*_*path*, *best*_*config* = *get*_*max*_*acc*_*model*(*result*);

**end**

**Function** *trainmodel*(*replicated*_*layers*, *path*):

 *model* = *createmodel*(*replicated*_*layers*)

 **if** *path* **then**

  *previous*_*model* = *load*_*model*(*path*);

  *model* = *weight*_*transfer*(*previous*_*model*, *model*);

 **end**

 train *model*, return (*accuracy*, *path*);

### 3.7 Control variables

- **threshold**: The threshold value for the accuracy improvement check. It is used to determine when to stop the replication rounds.
- **max_acc**: The maximum accuracy observed so far. It is updated whenever a better accuracy is found.
- **max_acc_in_rep_round**: The maximum accuracy achieved in the current replication round. This variable helps in determining whether the current round has yielded a better configuration.
- **allow_replicate_layers**: A list of layers that are allowed to be replicated. This controls which layers in the model can be modified during the search process.
- **best_replicated_config**: The best configuration of replicated layers found so far. It is used as the starting point for the next round of replication.
- **best_model_path**: The file path of the model that achieved the best accuracy. This path is also used for loading and transferring weights to new models.
- **config_gpu0, config_gpu1, config_gpu2, config_gpu3, config_gpu0_ssh**: Specific configurations for different GPUs used in concurrent training. These configurations determine how the layers are replicated and distributed across GPUs. Since in the concurrent training, one GPU handles one type of configuration, this specifies which GPU replicates which layer replications in the configuration.
- **command_run**: A list of commands to execute concurrent training on different GPUs. This list includes training commands for all specified configurations. It can be shell commands that need to be invoked according to the machine setup.

## 4 Experimental results

The experimental results presented in this paper include the following components:

1. Performance comparison between 2D and 3D MobileNetV2 models in both original and distributed training settings. This includes evaluating the differences in model performance in terms of accuracy and other relevant metrics.
2. The results of automatic model finding on distributed training
3. The results of automatic model finding on concurrent training
4. A comparative analysis of the performance of the automatic model finding on distributed training and concurrent training

### 4.1 Case 1: Distributed training

We compare the performance of MobileNetV2 models based on three different types of input data: RGB, RGBD, and RGB+D.

For the case of RGB, we assume the baseline as MobileNetV2 without depth information and we searched for the good replication configuration. For RGBD and RGB+D, depth map was integrated in two different ways as described in Sections 3.4.2 and 3.4.3. This is the approach used in [1]. In RGB (DDP), RGBD (DDP), and RGB+D (DDP), distributed training with data parallel was utilized for 4 GPUs.

In Table 1, MobileNetV2 with RGB+D input data achieves the highest accuracy at 92.35%, followed by the RGBD input data at 88.96%, and the RGB (2D) input data results in the lowest accuracy at 86.4%. The result suggests that adding 3D data as input to the model improves its accuracy. The RGBD input increases the depth (input channels) from 3 to 4, allowing the model to learn features from the additional data. However, the RGB+D input, consisting of separate processing for RGB and depth (D) data before merging, leads to the highest accuracy. This separate processing of RGB and depth data before entering the model contributes to the superior performance of the RGB+D input.

**Table 1:** Performance comparison between each model condition.

Model	*Accuracy*	Time(minutes)	Images/sec	parameters
RGB	86.4%	1,200.09	140	3,761,072
RGBD	88.96%	1,021.22	125	3,761,360
RGB+D	**92.35**%	1,232.09	130	3,761,424
RGB (DDP)	86.22%	300.00	950	3,761,072
RGBD (DDP)	88.05%	324.39	829	3,761,360
RGB+D (DDP)	**92.28**%	320.82	816	3,761,424

When using distributed training, it is noticed that the training throughput was increased about 7 times and the training time was reduced almost by 1/4. Due to the nature of computing, the distributed training has the tradeoff on accuracy and speed [19]. One must explore the proper batch size for each task to lead to the good accuracy. Several approaches can be applied to improve the accuracy for distributed training such as changing the optimizer, learning rate warmup, etc.

From the experimental results, training the model on a single GPU and training the model using distributed data parallelism (DDP) yielded similar accuracy results, with normal training achieving slightly higher accuracy in some cases. Specifically, normal training for RGB+D achieved 92.35%, while DDP training for RGB+D achieved 92.28%.

However, it is important to highlight that the primary benefit of DDP training lies in its significant reduction of training time. The DDP approach reduces the training duration by up to 75%, allowing for a substantial increase in the number of images processed per second. This efficiency gain is crucial for practical applications where training time is a significant constraint. For instance, the training time for RGB+D (DDP) was reduced to 320.82 minutes compared to 1,232.09 minutes for normal training. The improved training efficiency makes DDP training advantageous despite the marginal difference in accuracy.

The accuracy of 92.28% for RGB+D (DDP) was highlighted as the best because it represents the highest accuracy achieved with the distributed training method, which balances both accuracy and training efficiency. The slight difference between 92.35% (RGB+D normal training) and 92.28% (RGB+D DDP training) is negligible when considering the significant reduction in training time and computational resources required.

### 4.2 The results of automatic model finding on distributed training

**Fig 16:**
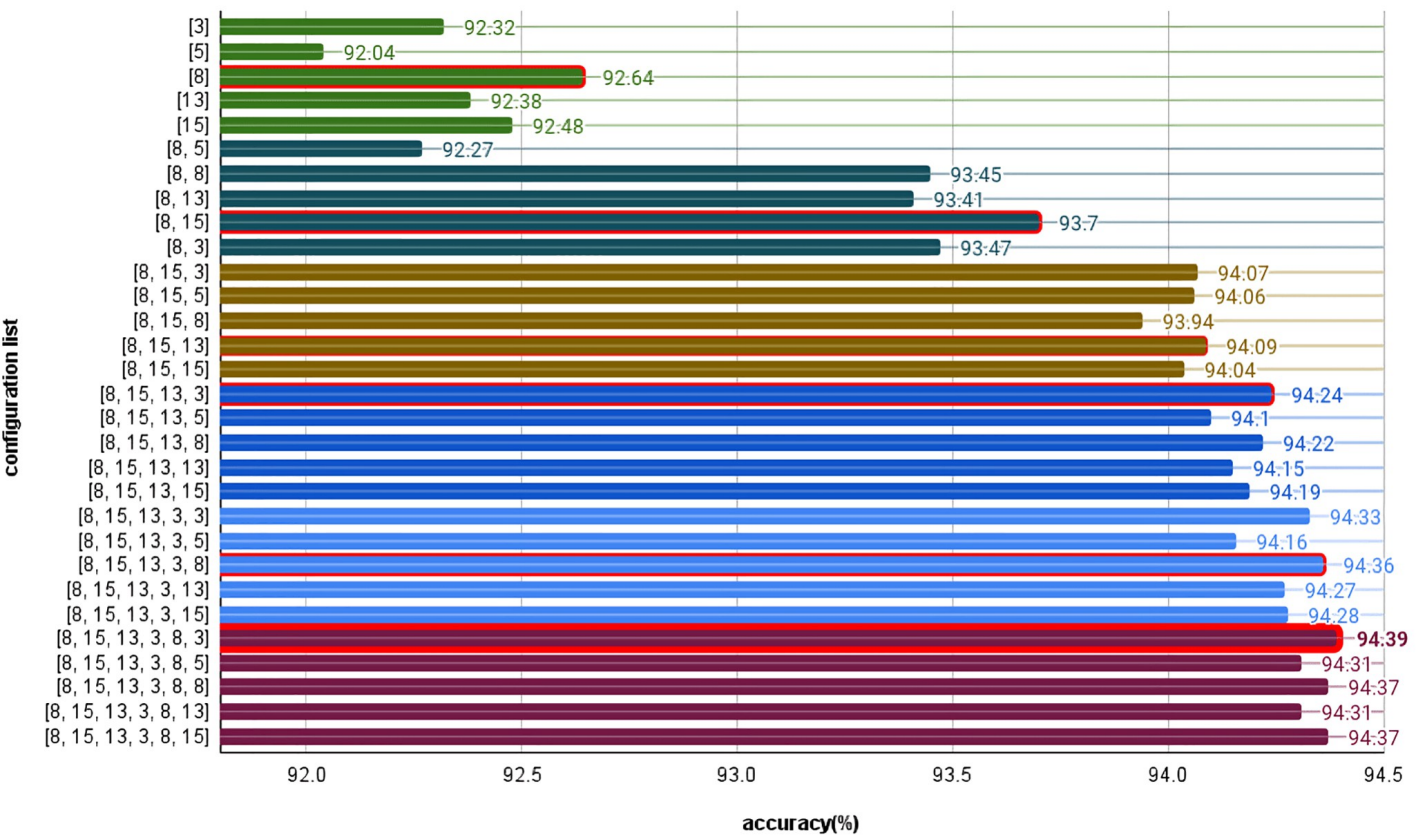
Result of automatic model finding on distributed training.

The experimental results of automatic model findings on distributed training show that this process can significantly enhance the model’s performance when learning from the same dataset. When starting from the best layer replication configuration for the first layer ([8] -> [8, 15]), we achieved the most significant improvement of 1.06% for accuracy. As the layer replication proceeds from layer two ([8, 15] -> [8, 15, 13]) to layer three ([8, 15, 13] -> [8, 15, 13, 3]), the improvement gradually decreases until it eventually stops, suggesting that the transfer of weights between the original and expanded models, except for the expanded layer, is vital for significant learning improvement. The number of weight transfers into highly expanded layers has a diminishing effect on performance improvement, which eventually leads to the stopping of the search process when the accuracy improvement falls below the specified stopping threshold (0.1), as shown in Fig 16.

### 4.3 Case 2: Concurrent training

When automatically searching for models with layer replication during concurrent training under various conditions, the best layer replication positions found are as follows: In the first iteration, position [5] on GPU1, Server1, achieved the highest accuracy of 92.26%. In the second iteration of layer replication, positions [5, 15] on GPU0, Server2, achieved the highest accuracy of 93.96%. In the third iteration, position [5, 15, 15] on GPU0, Server2, achieved the highest accuracy of 94.51%. In the fourth round of layer replication, position [5, 15, 15, 13] on GPU3, Server1, achieved the highest accuracy of 94.76%. In the fifth iteration, positions [5, 15, 15, 13, 5] on GPU1, Server1, achieved the highest accuracy of 94.92%. In the final iteration, positions [5, 15, 15, 13, 5, 8] on GPU2, Server1, achieved the highest accuracy of 94.97%.

**Fig 17:**
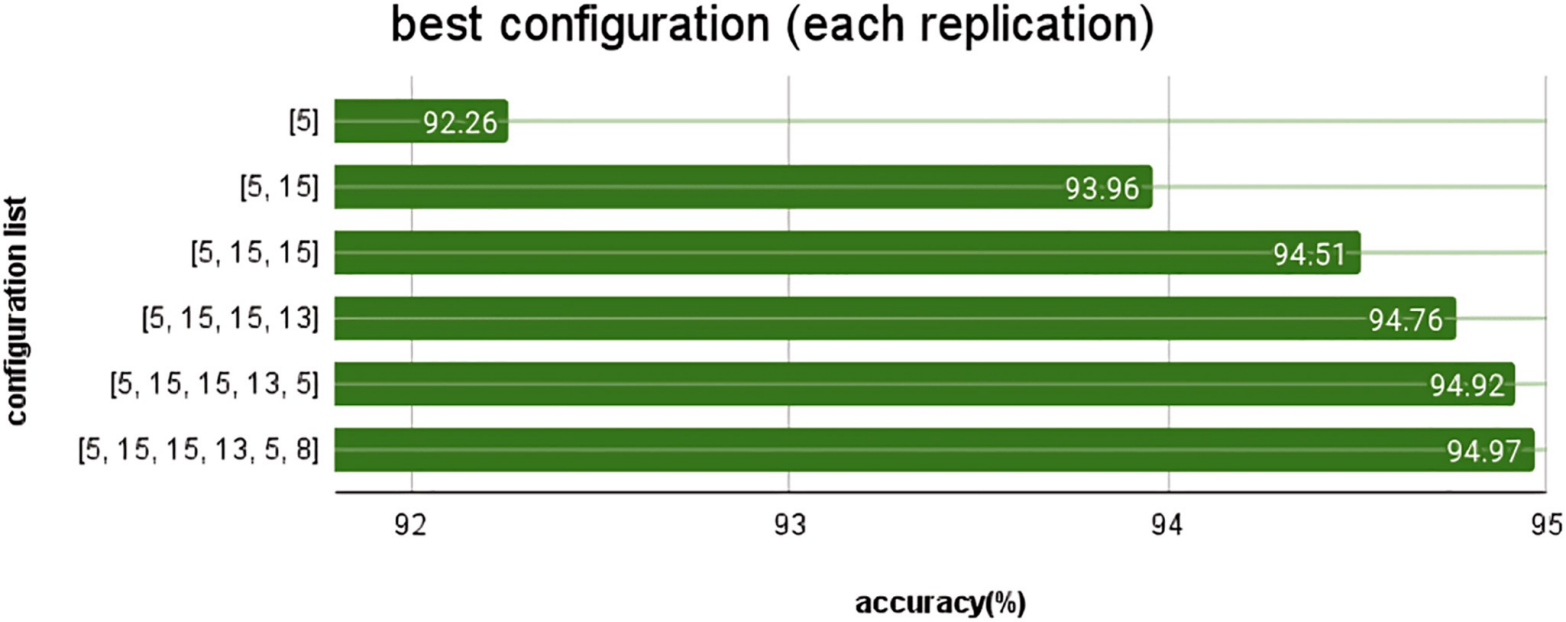
Result of the best layer replication positions.

These results are shown in Fig 17, illustrating the positions with the highest accuracy during the automatic layer replication process in different configuration training.

**Fig 18:**
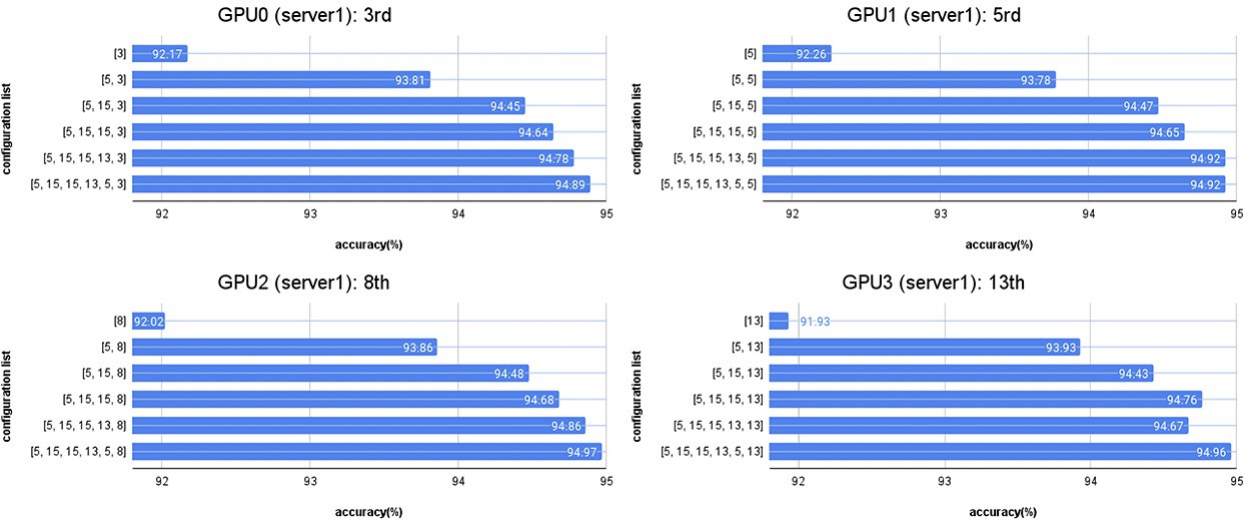
Results of the best layer replication position in Server1.

**Fig 19:**
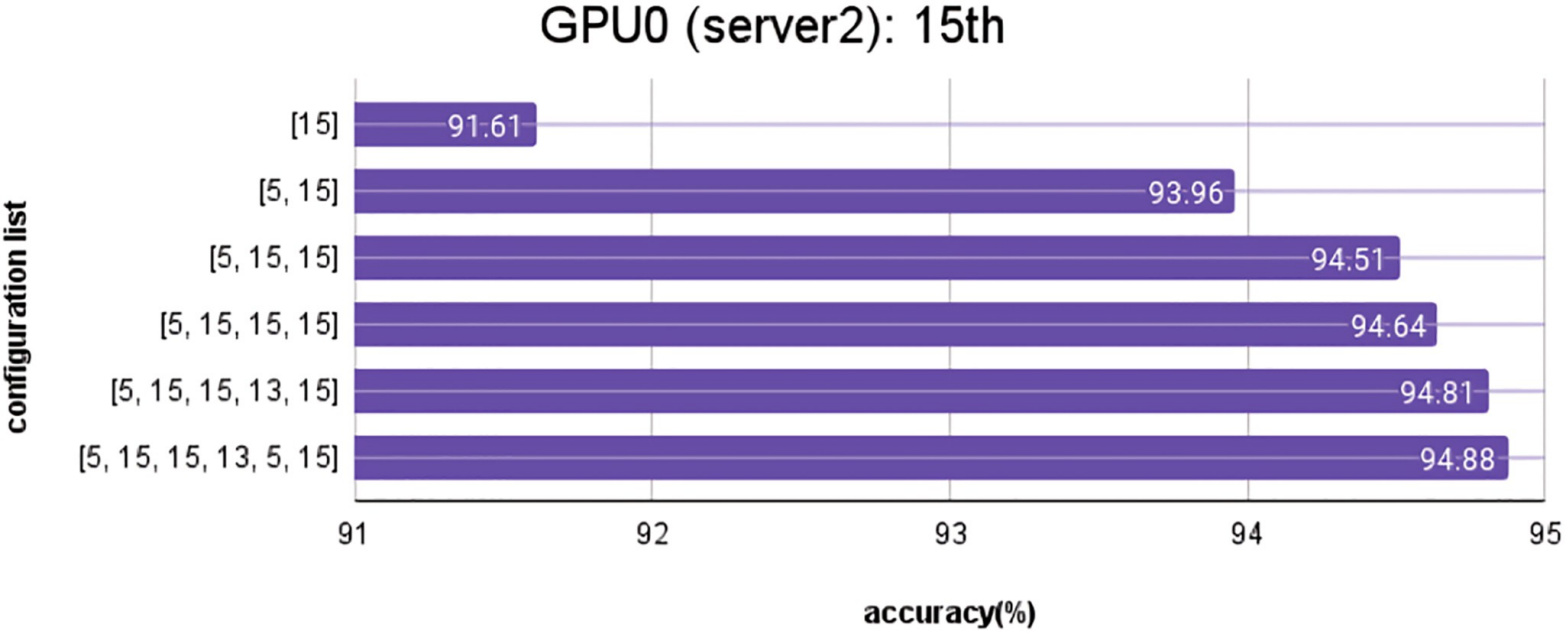
Results of the best layer replication position in Server2.

From Fig 18, the results show the layer replication process on Server 1 with 4 GPUs. Model training with layer replication at position 3 was performed on GPU0, model training with layer replication at position 5 on GPU1, model training with layer replication at position 8 on GPU2, and model training with layer replication at position 13 on GPU3. However, it was not possible to train the model with layer replication at position 15 on Server 1 due to an insufficient number of GPUs. Therefore, model training with layer replication at position 13 had to be carried out on Server 2, which has 2 GPUs, as shown in Fig 19.

### 4.4 A comparative analysis of the performance of the automatic model finding on distributed training and concurrent training

From Table 2, it can be observed that the accuracy of both automatic model search methods is approximately the same. However, training the model concurrently has a slightly higher accuracy, likely due to differences in batch size and learning rate. Additionally, the stability of the connections between GPUs during distributed training affects the model’s accuracy and the time required for automatic model search on concurrent training is less than on distributed data training. This is because searching for models on distributed data training involves training the model in each sequential layer replication, which takes more time compared to searching for models on concurrent training, even though distributed data training is faster than the original training method.

**Table 2:** Performance comparison between distributed training and concurrent training.

Type	config	Accuracy	Time(minutes)
Distributed training	[8, 15, 13, 3, 8, 3]	94.39%	5,827.72
Concurrent training	[5, 15, 15, 13, 5, 8]	94.97%	3,888.00

The total number of parameters in the model after layer replication is about the same as the number of parameters before replication since the model is expanded using the same block connected to the same input and output size block. Therefore, the number of parameters does not increase.

Comparing to [2], in Table 1, when considering automatic model finding, the solutions obtained have higher accuracy as presented in Table 2.

### 4.5 Discussion

One of the primary concerns when replicating layers in the MobileNet architecture is the potential increase in overall training time. Our method indeed introduces additional complexity, which could impact training duration. However, several factors justify this trade-off:

1. **Accuracy vs. Training Time Trade-off**:
  - **Accuracy Improvement**: As demonstrated in our experiments, the proposed method improves the model’s accuracy by up to 6% compared to previous work on 3D MobileNetV2 and by 8% compared to the vanilla MobileNetV2. In many practical applications, even small increases in accuracy can lead to significantly better performance, especially in critical tasks like 3D face recognition where precision is paramount.
  - **Use Case Importance**: In contexts such as security, healthcare, and augmented reality, where 3D face recognition is critical, the benefits of higher accuracy often outweigh the additional computational cost. The enhanced accuracy can lead to more reliable and robust systems, reducing the risk of errors and improving user experience.
2. **Efficiency of Training Methods**:
  - **Distributed Data-Parallel Training**: By utilizing distributed data-parallel training across multiple GPUs, we have significantly mitigated the increased training time. Our results show a reduction in training time by up to 75% compared to traditional single-GPU training. This demonstrates that with proper resource allocation, the additional complexity does not necessarily translate to prohibitively longer training times.
  - **Concurrent Training**: Our concurrent training approach further accelerates the training process, achieving an additional time saving of 1,932 minutes compared to distributed training. This indicates that the training time can be managed effectively even with increased complexity.
3. **Scalability and Future Improvements**:
  - **Scalability**: The approach we propose is scalable. As more computational resources become available, the training time can be further reduced, making the layer replication strategy more feasible.
  - **Improvement**: More efficient training strategies and optimizations can be considered to further reduce the training time. For instance, techniques like gradient checkpointing, model pruning, and mixed-precision training could be employed to balance accuracy improvements with training efficiency.

## 5 Conclusion

We propose an approach to enhance the model architecture search based on MobileNetV2. Our approach targets at two aspects: the use of automatic layers modification and distributed training. In the first aspect, we consider the layer replication as an operator to improve the accuracy and attempt to find the proper layers to replicate and weight transfers. The heuristic to find the proper configuration is proposed. Secondly, the distributed training with data parallel and concurrent model training were utilized to speed up the model training time and the configuration searching.

The derived model achieved up to 6% higher accuracy compared to previous work on 3D MobileNetV2 and 8% higher accuracy compared to the vanilla MobileNetV2.

The use of distributed data-parallel training across four GPUs reduced the model training time by up to 75% compared to traditional single-GPU training. Additionally, the concurrent training approach further reduced the time to find an optimal solution by 1,932 minutes compared to the distributed training approach.

Future work will focus on applying this technique to models with similar structures to MobileNetV2 and combining automatic model training using both distributed and concurrent training approaches to achieve even greater performance improvements. The techniques to improve the distributed training can also be explored.
