# Correction: Accelerating automatic model finding with layer replications case study of MobileNetV2

**DOI:** 10.1371/journal.pone.0329645

**Published:** 2025-08-04

**Authors:** Kritpawit Soongswang, Chantana Chantrapornchai

The Funding statement for this article is incorrect. The correct Funding statement is as follows: National Research Council of Thailand (NCRT, Project No. NRCT5-RSA63002-14). The funders had no role in study design, data collection and analysis, decision to publish, or preparation of the manuscript.
